# Genetic Algorithm-Optimized Volume Holographic Gratings in Ultra-Thin MiniLED Modules

**DOI:** 10.3390/mi17040479

**Published:** 2026-04-15

**Authors:** Zechao Shen, Yue Zhang, Guoqiang Lv, Zi Wang, Qibin Feng

**Affiliations:** 1 School of Instrument Science and Opto-Electronics Engineering, Hefei University of Technology, Hefei 230009, China; 2024170056@mail.hfut.edu.cn (Z.S.); 2024170079@mail.hfut.edu.cn (Y.Z.); guoqianglv@hfut.edu.cn (G.L.); 2 Special Display and Imaging Technology, Innovation Center of Anhui Province, Anhui Province Key Laboratory of Measuring Theory and Precision Instrument, Hefei University of Technology, Hefei 230009, China; wangzi@hfut.edu.cn

**Keywords:** volume holographic gratings, MiniLED, backlight module, genetic algorithm

## Abstract

The design of volume holographic gratings (VHGs) is traditionally based on monochromatic plane waves. However, practical applications often involve light sources with broad wavelength bandwidths and certain emission areas, such as LEDs and MiniLEDs, which cause significant Bragg mismatch and degrade diffraction efficiency. To address this fundamental challenge, this paper proposes a novel, to the best of our knowledge, genetic algorithm (GA)-based optimization method for VHG design. A ray-tracing analysis model that fully incorporates the spectral and spatial characteristics of extended broadband sources is established. The GA optimizes the grating fabrication angles by minimizing a fitness function defined as the residual energy after diffraction, thereby achieving optimal performance under non-ideal illumination conditions. The effectiveness of the proposed method is demonstrated through a case study: suppressing the high-intensity central beam in an ultra-thin MiniLED backlight module (BLM). Simulation and experimental results show that the GA-optimized VHG significantly reduces the peak irradiance from 5.01 W/cm^2^ to 4.14 W/cm^2^ at an optical distance (OD) of 0.5 mm. This work provides a robust and source-adaptive design methodology for VHGs, with potential applications extending beyond backlighting to areas such as augmented reality, holographic displays, and optical communications.

## 1. Introduction

Volume holographic gratings (VHGs) have garnered significant attention in advanced optical systems due to their unique multiplexing capabilities, high diffraction efficiency, and exceptional angular and spectral selectivity [1,2,3]. Based on the principles of Bragg diffraction, VHGs modulate light within a thick photosensitive medium, enabling complex wave-front engineering. Consequently, they have been widely deployed in applications ranging from augmented reality (AR) waveguide displays and head-up displays (HUDs) to spectral beam combining and optical communications [4,5]. However, the design and optimization of VHGs are traditionally based on ideal light sources, such as collimated monochromatic laser beams, which can be accurately described by Kogelnik’s coupled-wave theory [6]. When transitioning to practical illumination systems, the light sources (such as LEDs or MiniLEDs) are inherently non-ideal—these extended broadband sources possess a certain emission area, diverse spatial intensity distributions, and specific wavelength bandwidths [7]. Modulating such highly divergent and non-monochromatic light fields using VHGs presents a formidable challenge, as the diffraction behavior becomes a highly non-linear, multi-variable problem where traditional analytical methods fail to provide optimal structural parameters.

To overcome this limitation, computational optimization methods have been introduced into complex optical engineering [8,9]. Among various techniques such as particle swarm optimization (PSO), simulated annealing (SA), and direct search methods [10,11], genetic algorithms (GA) demonstrate superior performance in high-dimensional problems [12,13,14,15]. Given that VHG optimization involves multiple fabrication angles and nonlinear coupling between spectral and angular domains, GA is a well-suited choice. In this paper, we propose a comprehensive VHG design methodology tailored for non-ideal, extended light sources. We establish a 3D angular discretization model to accurately map the spatial emission characteristics to the Bragg diffraction conditions, followed by GA-based global optimization of the grating fabrication parameters. To rigorously validate this methodology, we select the ultra-thin MiniLED backlight as a proof-of-concept application scenario, demonstrating that non-ideal MiniLED emission can be efficiently reshaped at a near-zero OD.

VHG offers a fundamentally different mechanism for the MiniLED backlight module (BLM). VHGs leverage the angular selectivity of Bragg diffraction to deflect nearly vertical central beams toward larger angles, with performance depending solely on incident angle and wavelength—enabling near-zero OD operation. Crucially, since VHGs are fabricated with collimated light, their optical properties are uniform across the film, inherently eliminating alignment requirements. This breaks the fundamental trade-off between achieving a low OD and maintaining strict alignment tolerance that plagues conventional microstructure films [16].

## 2. Analysis and Comparison of VHG Types for BLM

### 2.1. Structure and Principle of the VHG-Based BLM

**Figure 1:**
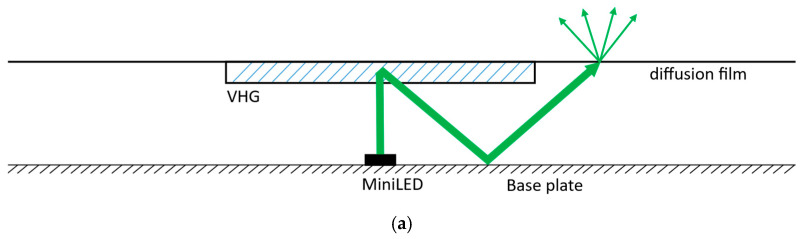

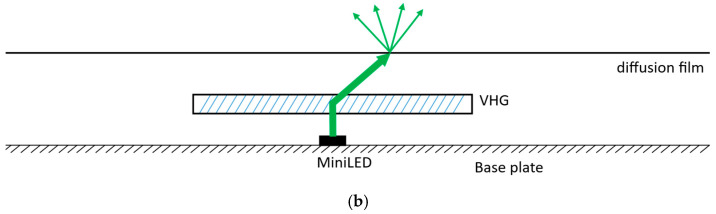
Schematic diagram of the VHG-based BLM. (**a**) BLM with reflection VHG; (**b**) BLM with transmission VHG.

Although the method is general, we first select the appropriate VHG type for the backlight case study. VHG is an optical component fabricated by processing photosensitive materials using interferometric exposure technology. This process involves recording the interference pattern formed by two coherent light beams, which induces a photochemical reaction in the photosensitive material at the bright fringe locations. This creates a periodic distribution of refractive index modulation, which enables the modulation of light beams. VHGs are classified as transmission or reflection based on whether the two coherent beams are incident on the same side or opposite sides of the photosensitive material. This paper first introduces the application of two types of gratings in backlighting, followed by a comparative analysis to decide which is more suitable for application in ultra-thin BLM. The BLMs with different kinds of VHG are illustrated in Figure 1 below, where the diffuser film can also be replaced with films with designed microstructures.

As illustrated in Figure 1, both reflection and transmission VHGs can deflect the central beam toward larger angles. In Figure 1a, the reflection VHG diffracts the incident beams toward the base plate, where they are reflected by a high-reflectivity film attached to the base plate. In Figure 1b, the transmission VHG allows the incident beam to pass through and exit at large diffraction angles directly, enabling a more compact configuration. A deeper comparative analysis of these two VHG types is presented below to determine which is more suitable for ultra-thin MiniLED BLMs.

### 2.2. Performance Comparison of Reflection and Transmission VHGs

To better compare the suitability of transmission and reflection VHGs for ultra-thin BLMs, we first calculate their angular and wavelength bandwidths based on Kogelnik coupled-wave theory. For a lossless transmission VHG, the diffraction efficiency η is given by:

(1) $$\eta =\sin^{2}(\nu )$$

where ν is the coupling strength parameter, reflecting the grating’s ability to modulate incident energy. The formula for calculating ν is:

(2) $$\nu =\frac{\pi \cdot △n_{0}\cdot d}{\lambda_{0}\sqrt{\left|c_{R}\cdot c_{S}\right|}}$$

where $\Delta n_{0}$ is the refractive index modulation, d is the thickness of the grating medium, $\lambda_{0}$ is the optical wavelength under calculation, and $c_{R}$ and $c_{S}$ are the directional cosines of the reference and signal beams inside the medium, which are related to the recording angles, incident light wavelength, and refractive index of the waveguide. Since ν is proportional to ${\Delta n}_{0}$, the efficiency oscillates periodically with increasing modulation depth. The first maximum efficiency occurs when ν=π/2, yielding the optimal refractive index modulation given by:

(3) $$△n_{0}=\frac{\lambda \cdot \cos\theta_{0}}{2\cdot d}$$

where $\theta_{0}$ denotes the Bragg angle of the VHG. Substituting the parameters from Table 1 into Equation (3) gives $\Delta n_{0}=0.018$. Further increasing ${\Delta n}_{0}$ would cause over-modulation, coupling energy back to the 0th order and generating higher-order diffraction, thereby degrading performance. Therefore, the ${\Delta n}_{0}$ of transmission VHG is set to its theoretical optimal value 0.018. For a reflection VHG, the diffraction efficiency under the Bragg condition is:

(4) $$\eta_{2}=\tanh^{2}(\nu )$$

Unlike the transmission VHG, Equation (4) shows that $\eta_{2}$ increases monotonically with ${\Delta n}_{0}$ and saturates toward unity, with no over-modulation risk. Thus, to achieve maximum diffraction efficiency, Δn should be as high as the material permits. In this study, the ${\Delta n}_{0}$ of reflection VHG is set to 0.030, the upper limit achievable by the photosensitive material.

This configuration ensures that both types of gratings are compared under their respective optimal operating conditions, thereby enhancing the reference value of subsequent analyses on wavelength and angular bandwidth.

**Figure 2:**
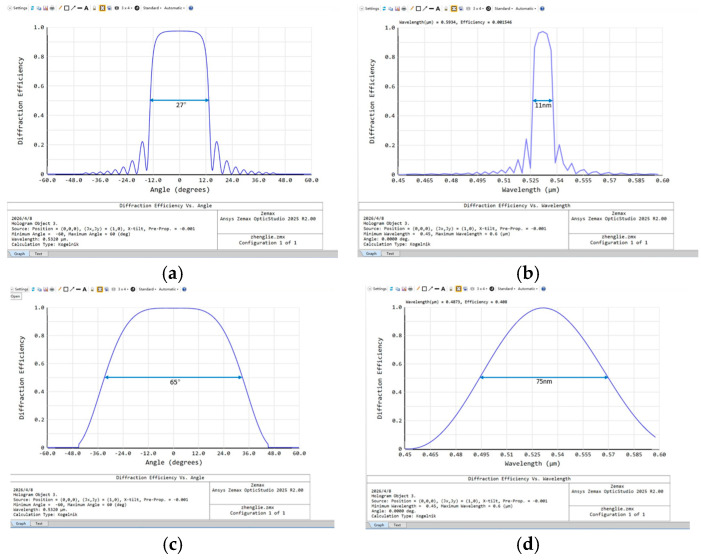
Performance comparison between transmission and reflection VHGs. (**a**) Angular selectivity curve of the reflection VHG; (**b**) Wavelength selectivity curve of the reflection VHG; (**c**) Angular selectivity curve of the transmission VHG; (**d**) Wavelength selectivity curve of the transmission VHG.

The calculation results are shown in Figure 2, where transmission VHG exhibits broader wavelength and angular bandwidth. In contrast, although the reflection VHG demonstrates superior angular selectivity, its wavelength bandwidth is extremely narrow. Considering that the MiniLED light source has a full width at half maximum (FWHM) of about 40 nm, the reflection VHG cannot fully cover the emission spectrum of the MiniLED. Therefore, the central beam modulation element must have wide wavelength coverage to prevent diffraction efficiency degradation caused by wavelength mismatch. Based on this requirement, this paper selects the transmission VHG as the central beam modulation element for BLM.

## 3. GA-Based Design Methodology of Transmission VHG

MiniLED is a kind of light source with a certain emission area and wavelength bandwidth. Its emission spectrum is considerably broader than the monochromatic laser wavelength used for VHG fabrication, leading to significant wavelength mismatch during grating reconstruction, which in turn affects diffraction efficiency and the suppression of the high-intensity central beam. To effectively compensate for Bragg mismatch introduced by the broad spectrum and extended source characteristics, this paper introduces the GA for global optimization of the VHG fabrication parameters. GA is an optimization method that simulates natural selection and genetic mechanisms, making it suitable for solving complex multi-variable, nonlinear optimization problems. To implement GA-based optimization of the fabrication angles, a ray-tracing-based analysis model with the consideration of broadband and extended light sources is first established. This model accurately describes the light field distribution of MiniLED in the spatial, angular, and spectral domains. Subsequently, with the residual light intensity in the central region as the optimization objective, GA iteratively searches for the optimal fabrication angles, which enables efficient deflection of central beams even under broadband extended source conditions.

### 3.1. Ray-Tracing-Based Analysis Model

To accurately evaluate VHG diffraction performance under non-ideal conditions, a numerical simulation model based on angular discretization is established. Unlike the monochromatic laser used for VHG fabrication, the MiniLED light source has a broad wavelength range and a certain emission area. To address this complex scenario, the model integrates a ray-tracing algorithm with three-dimensional Bragg mismatch calculations based on vector analysis. By performing weighted integration of Kogelnik coupled-wave theory across the spatial, angular, and wavelength domains, the model quantifies efficiency attenuation caused by wavelength mismatch and angular deviation. This provides a theoretical foundation for optimizing the grating fabrication parameters.

**Figure 3:**
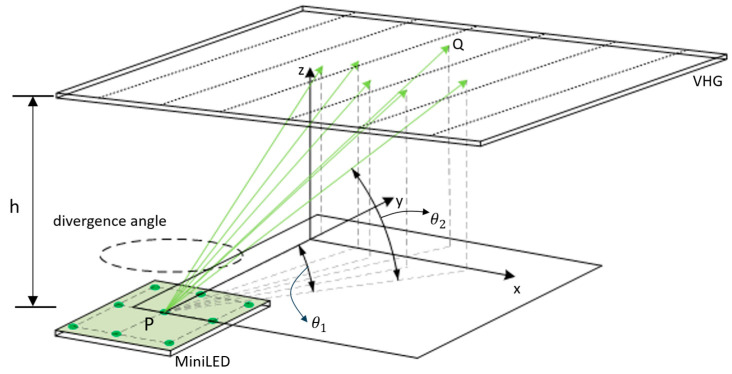
Ray-tracing-based analysis model with the consideration of broadband and extended light sources.

As shown in Figure 3, the emitted beam is denoted as $F(\theta_{1},\theta_{2})(x,y)$, where F represents the energy carried by the beam, and (x,y) are the coordinates of the emitting point P. The angles $\theta_{1}$ and $\theta_{2}$ define the direction of the beam: $\theta_{1}$ is the angle between the projection of the beam onto the xy-plane and the y-axis, while $\theta_{2}$ is the angle between the projection onto the zy-plane and the y-axis. Here, h denotes the distance from the MiniLED to the VHG. The MiniLED chip is discretized into a cluster of point light sources, denoted as $P_{i}$. For each point source, the luminous angular range is further discretized into a set of discrete angles. Based on the intersection points of the beams with the grating plane, the points Q are generated. Through this approach, each emitting point P is associated with a corresponding set of Q points. For transmission VHG, the diffraction efficiency $\eta (P,\theta_{1},\theta_{2},\lambda )$ for a single monochromatic beam is given by:

(5) $$\eta \left(P,\theta_{1},\theta_{2},\lambda \right)=\frac{\sin^{2}(\sqrt{\nu^{2}+\xi^{2}})}{1+\xi^{2}/\nu^{2}}$$

where ξ is the Bragg mismatch parameter, representing the degree to which the beam deviates from the Bragg condition. The formula for calculating ξ is:

(6) $$\xi =\frac{\delta \cdot d}{2c_{S}}$$

The corresponding deviation factor δ in Equation (6) is given by:

(7) $$\delta =\Delta \theta \cdot K\sin(\Phi -\theta_{ref})-\frac{\Delta \lambda \cdot K^{2}}{4\pi n}$$

where Δθ is the offset between the reconstruction beam incidence angle and the reference beam incidence angle. Δλ is the offset between the current reconstruction wavelength and the reference wavelength. K is the grating vector magnitude, defined as K=2π/Λ, where Λ is the grating period. Φ is the slant angle of the grating. $\theta_{ref}$ is the incident angle of the reference beam inside the medium during VHG fabrication. For a single beam with a certain wavelength, the energy transmitted as the 0th order beam through the VHG is given by:

(8) $$R\left(P,\theta_{1},\theta_{2},\lambda \right)=[1-\eta \left(P,\theta_{1},\theta_{2},\lambda \right)]\cos\theta_{2}\cdot S(\lambda )$$

where S(λ) is the spectral power distribution of the light source, and $\cos\theta_{2}$ is the cosine intensity distribution of the Lambertian source.

Given that the MiniLED chip has a certain emission area, certain divergence angle range, and certain wavelength, the final central residual energy E of the system can be obtained by performing several numerical integrations over the spatial grid P, the angular distribution $\left(\theta_{1},\theta_{2}\right)$, and the spectral distribution λ.

(9) $$E=\int_{P_{0}}^{P_{N}}\int_{\theta_{1\min}}^{\theta_{1\max}}\int_{\theta_{2\min}}^{\theta_{2\max}}\int_{\lambda_{\min}}^{\lambda_{\max}}R\left(P,\theta_{1},\theta_{2},\lambda \right)$$

It should be noted that the above ray-tracing model assumes unpolarized light, which is consistent with the actual MiniLED emission. In Kogelnik’s coupled-wave theory, the diffraction efficiency for transmission VHG depends on the polarization state via the coupling strength parameter ν. However, for the unpolarized case, the effective efficiency is the average of the TE and TM responses. Since the directional cosines $c_{R}$ and $c_{S}$ used in our model are purely geometric quantities derived from the grating vector and the incident wavevector, they do not intrinsically distinguish between polarizations. Therefore, the calculated efficiency for each beam implicitly represents the average over polarization. This simplification is widely accepted for engineering applications involving unpolarized light sources such as MiniLEDs [17].

### 3.2. GA-Based Optimization Design of Transmission VHG

As established by the physical model and ray-tracing process for the MiniLED backlight system, the fabrication angles $\left(\theta_{ref},\theta_{obj}\right)$ are critical. Here, $\theta_{ref}$ and $\theta_{obj}$ denote the incident angles of the reference and object beams during VHG fabrication, respectively. These angles directly govern the grating vector and thus the Bragg matching performance of the VHG. The wavelength distribution and angular emission profile of MiniLEDs are nonlinearly coupled, with the consideration of their broad wavelength range and certain emission area. This makes traditional single-point Bragg matching inadequate for achieving global optimization. Therefore, this paper combines the previously established analysis model with a GA to minimize the intensity of centrally transmitted light by optimizing the fabrication angles.

To implement GA-based optimization of the fabrication angles, a fitness function must first be defined. The central residual energy E derived in Equation (9) is adopted as the fitness function, with the consideration of the broad wavelength range and certain emission area of MiniLEDs, i.e., F=E. The optimization variables are the fabrication angles $\left(\theta_{ref},\theta_{obj}\right)$, with a search range of −60° to 60°. The objective is to minimize E. During the evolutionary process, angle combinations yielding lower transmission energy have a higher probability of being selected for the next generation [18].

Furthermore, Equation (7) reveals that the wavelength mismatch term is proportional to the square of the grating vector K. When $\theta_{ref}$ and $\theta_{obj}$ are close, the grating vector K approaches zero, causing the wavelength mismatch term to become ineffective. This would lead to clearly suboptimal results. Therefore, based on the required deflection angle for the BLM, this paper imposes a constraint: the difference between $\theta_{ref}$ and $\theta_{obj}$ must be larger than 45°. Within this model, the optimization objective is to find a set of optimal fabrication parameters that minimize the value of the fitness function. This implies that the VHG can maximally reduce the vertically transmitted light, with the consideration of both spectral shift and source size, thereby shifting energy from the central region to the periphery.

## 4. Simulation and Experiment

### 4.1. Simulation Verification

**Figure 4:**
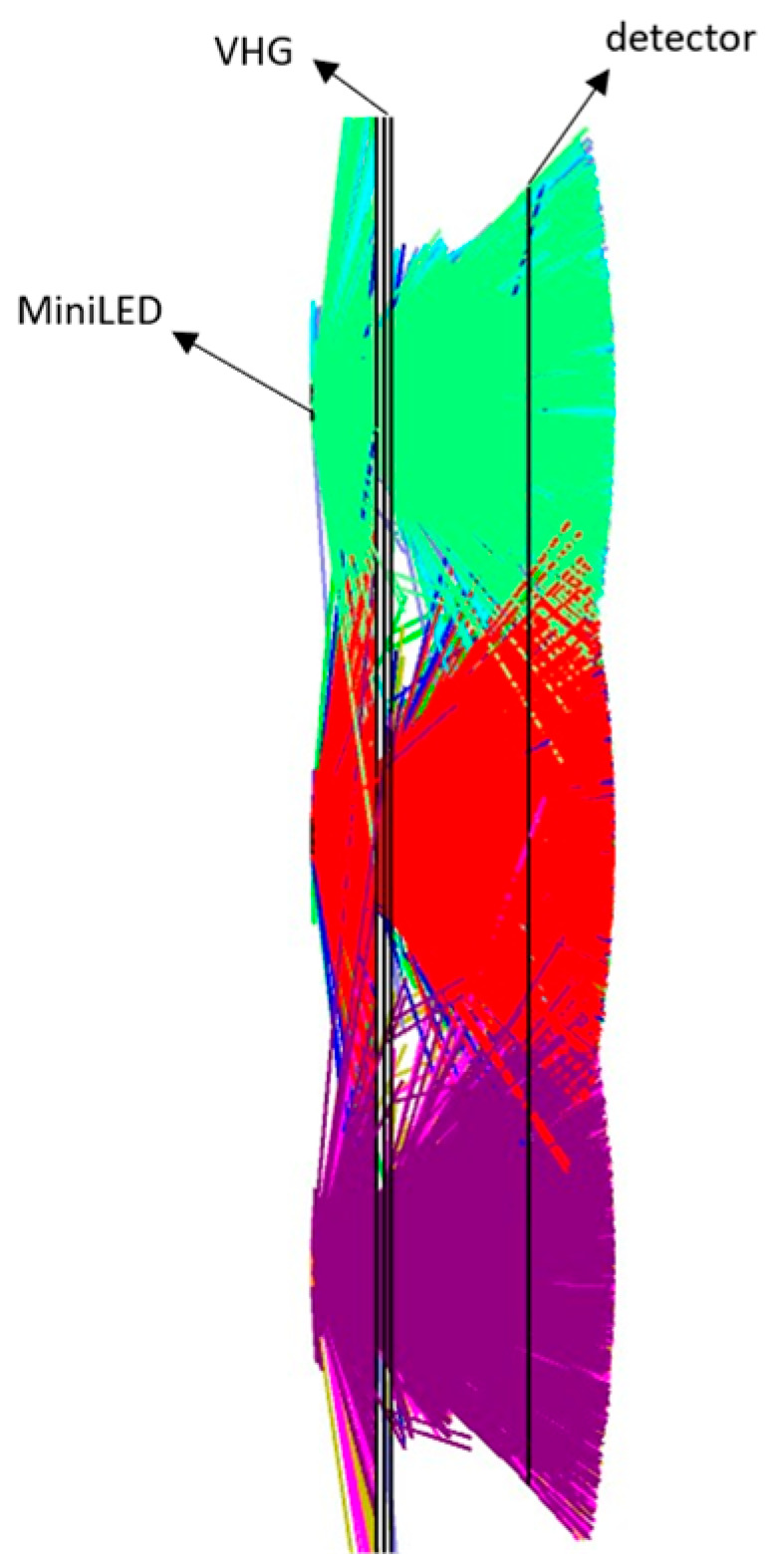
Ray tracing diagram of the transmission VHG optical film.

In practical full-colour MiniLED backlights, the common configuration uses blue chips combined with a quantum dot film to generate white light. The blue chip has a certain spectral bandwidth, and the VHG is designed for a single wavelength to work with the quantum dot film. However, due to two practical limitations in our laboratory. First, the available MiniLED backlight panel uses green chips instead of blue chips. Second, our existing single-longitudinal-mode laser is at 532 nm, whereas the corresponding 457 nm blue laser is a multi-longitudinal-mode laser, which leads to extremely poor VHG fabrication quality. Therefore, for the experimental validation of the proposed design methodology, we used the green MiniLED. To validate the effectiveness of the proposed algorithm, a simulation model for a BLM with the transmission VHG is established, as shown in Figure 4. In the model, the MiniLED chip size is 0.508 mm × 0.508 mm; the distance between the VHG and the light source substrate is set to 1.0 mm; the pixel distance of MiniLEDs is 6.0 mm; the distance between the detector and the light source substrate is 3 mm; and the number of MiniLED chips is 3 × 3. The fabrication wavelength of the VHG in the model is 532 nm, with a refractive index modulation of 0.018.

**Figure 5:**
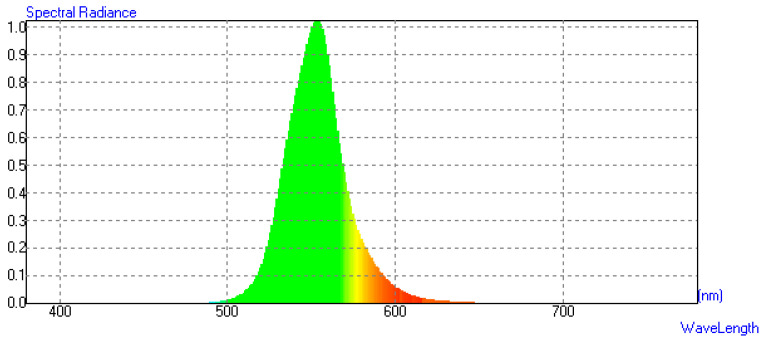
Spectral distribution of the MiniLED light source.

The spectral distribution of the MiniLED light source used in this study was measured using an SR-UL1R spectroradiometer (Topcon Company, Tokyo, Japan), as shown in Figure 5.

**Figure 6:**
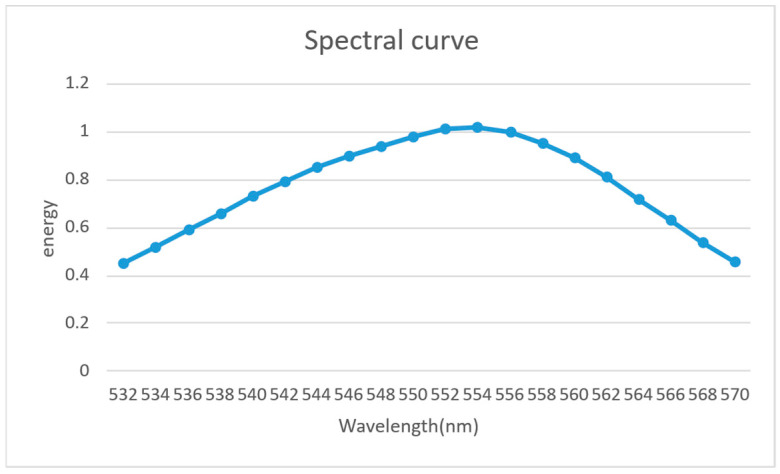
Spectral FWHM of the MiniLED light source.

Due to the limited number of wavelengths supported by the simulation software, the FWHM of the MiniLED wavelength distribution shown in Figure 5 is selected as the simulation wavelengths, with the distribution illustrated in Figure 6.

**Figure 7:**
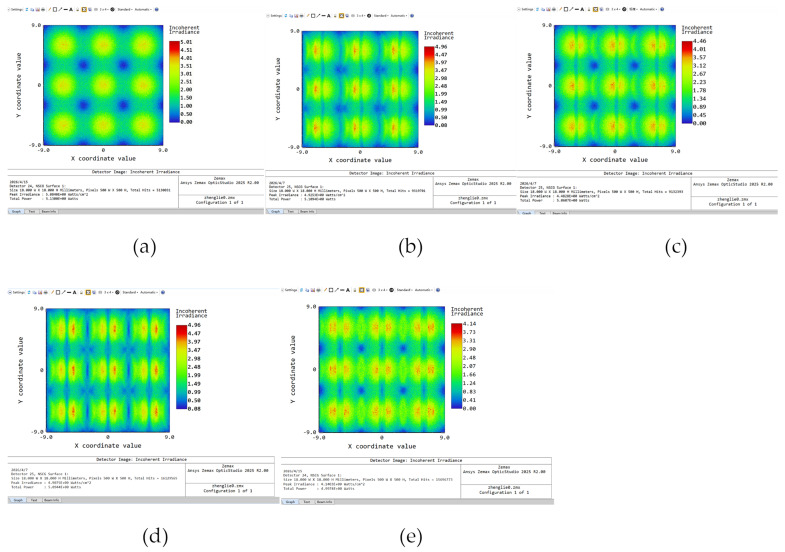
Illuminance distribution of the five simulation groups. (**a**) Without VHG; (**b**) With uncompensated VHG; (**c**) With GA-compensated VHG; (**d**) With dual-layer uncompensated VHG; (**e**) With dual-layer GA-compensated VHG.

To demonstrate the advantages of the GA-based design proposed in this paper, five sets of simulation experiments were conducted, with the consideration of the broad wavelength range and extended emission area of MiniLEDs. Group 1 is defined as the configuration without VHG. Group 2 is with the uncompensated VHG. Group 3 is with GA-compensated VHG. The simulation angles are listed in Table 2. The simulated illuminance distributions are presented in Figure 7a–c.

As shown in Figure 7a, the illuminance distribution without VHG exhibits a high-intensity central beam directly above the chip. As shown in Figure 7b, using only an uncompensated VHG has a very limited effect on attenuating the central light intensity. From the incoherent irradiance distribution, it can be observed that the reduction in peak intensity is marginal. Although dark bands are visible, the suppression at the center point is weak due to the influence of wavelength shift and the extended light source, and the dark bands exhibit noticeable curvature. From Figure 7c, it can be seen that the compensated VHG significantly suppresses the peak intensity, with the maximum irradiance decreasing from 5.01 W/cm^2^ to 4.46 W/cm^2^, while the dark bands are completely corrected.

However, the VHG-induced angular deflection leads to asymmetry in the intensity distribution, with the intensity on the left side significantly weaker than on the right side. The paper introduces two additional configurations: Group 4 with symmetric double-layer uncompensated VHG, and Group 5 with symmetric double-layer GA-compensated VHG. The simulation parameters for two groups are listed in Table 3 and the simulated illuminance distributions are presented in Figure 7d,e.

As shown in Figure 7d,e, the compensated VHG in the dual-layer structure further enhances the suppression of the central beam intensity, reducing the peak irradiance from 5.01 W/cm^2^ to 4.14 W/cm^2^ and further widening the central dark band. It should be noted that this result is the optimum obtained within the chosen design space: a single pair of fabrication angles and an OD of 0.5 mm. To further enhance the energy redistribution from the central region, future work may explore wavelength multiplexing to broaden the spectral response, or stacking more than two VHG layers, where each layer is recorded with a different pair of fabrication angles. These methods could redirect a larger fraction of the incident energy away from the central region, although they may increase fabrication complexity and introduce interlayer cross-talk.

### 4.2. Experimental Validation

**Figure 8:**
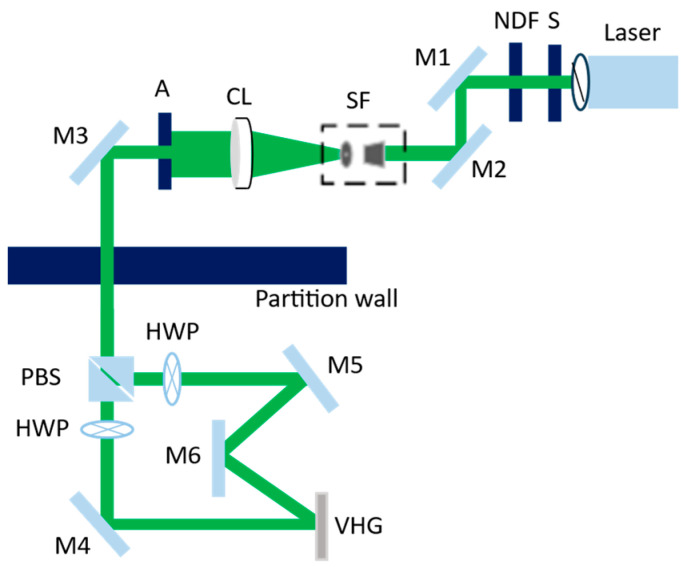
Optical setup for fabricating the transmission VHG.

Based on the simulation results, the GA-compensated dual-layer transmission VHGs were fabricated. The fundamental principle of VHG fabrication is to utilize two-beam laser interference to form a periodic refractive index modulation structure inside the holographic recording material. The interference exposure setup for the transmission VHG is shown in Figure 8:

In the figure, the laser is a single-longitudinal-mode laser with a wavelength of 532 nm, providing the coherent light source for holographic recording. S is a shutter used to control the exposure time precisely. A neutral density filter (NDF) adjusts the laser intensity to the desired level. Mirrors M1 and M2 are used to steer the beam and align it to the horizontal direction. The laser beam is then expanded and spatially filtered through a spatial filter (SF) and a collimating lens (CL) to obtain a clean, collimated beam. A (aperture) adjusts the beam size to match the required recording area. A polarizing beam splitter (PBS) divides the collimated beam into a reference beam and an object beam. A half-wave plate (HWP) is placed in one of the beams to adjust the intensity ratio and the polarization states of the object and reference beams, ensuring optimal interference contrast. The volume holographic material used in this work is Bayfol^®^ HX 200 (Covestro Deutschland AG, Leverkusen, Germany). Under 532 nm green light recording, this material exhibits a typical recording fluence of 20 mJ/cm^2^ and a refractive index modulation of 0.03. To avoid overmodulation caused by an excessively high refractive index modulation, we reduced the effective modulation by controlling the exposure dose. Through repeated experiments, an exposure dose of 6.7 mJ/cm^2^ was ultimately adopted. The measured diffraction efficiency of the transmissive VHG at the laser wavelength reaches 93.6%. The experimental results on MiniLED are as follows:

Experimental results show that the GA-compensated dual-layer VHG significantly suppresses the high-intensity central beam in MiniLEDs at an OD of 0.5 mm. Notably, owing to its large-area uniformity resulting from collimated light fabrication, the VHG eliminates the need for precise spatial alignment with the underlying light source. This is because the grating vectors are uniform across the entire film, making its optical response independent of lateral positioning relative to the chip array. This effectively overcomes the high alignment demands that challenge traditional micro-structured films in ultra-thin backlight applications.

**Figure 9:**
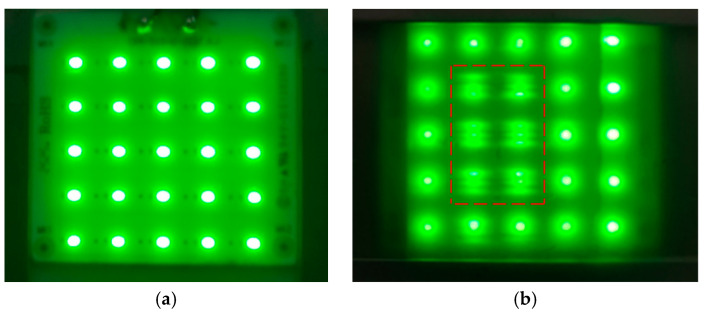
Experimental results. (**a**) A single-layer diffuser film placed at a height of 0.5 mm; (**b**) GA-compensated dual-layer VHG placed at a height of 0.5 mm.

The illuminance distributions presented in Figure 7 and Figure 9 serve as a primary proof-of-concept to demonstrate the VHG’s capability to deflect central beams at an extremely small OD. While the current uniformity does not yet meet the rigorous standards of commercial display products, these results represent a significant baseline achieved at near-zero OD. In traditional backlight units, a substantial physical mixing cavity is mandatory to suppress high-intensity central beams, which inherently limits the reduction in BLM thickness. It should be noted that most commercially available micro-structured optical films are designed and optimized specifically for the Lambertian distribution characteristics of conventional MiniLED light sources. However, after introducing the VHG layer in this study, the initial angular energy distribution of the BLM is reconfigured, resulting in a mismatch between the original optical films and the light field modulated by the VHG. Therefore, future work will focus on designing microstructure morphologies specifically tailored to the angular light distribution modulated by the VHG, aiming to fabricate an optimal BLM capable of achieving uniform light mixing at near-zero OD.

## 5. Conclusions

We have proposed and experimentally validated a GA-based design methodology for VHGs tailored to non-ideal extended broadband light sources. Unlike conventional designs assuming monochromatic plane waves, our method explicitly incorporates the wavelength bandwidth and the certain emission area of practical sources. A ray-tracing model discretizing the source in spatial and spectral domains is established, and the GA globally optimizes the fabrication angles by minimizing residual transmitted energy.

The methodology is demonstrated on an ultra-thin MiniLED BLM with an OD as low as 0.5 mm. Simulations show that the GA-optimized transmission VHG reduces the peak irradiance from 5.01 W/cm^2^ to 4.14 W/cm^2^ in a dual-layer configuration. Experiments using Bayfol^®^ HX 200 photopolymer at 532 nm with an optimized exposure dose of 6.7 mJ/cm^2^ confirm a diffraction efficiency of 93.6%. Because the VHG is recorded with collimated light, its optical response is uniform across the film, eliminating the need for precise alignment with the LED array.

Although demonstrated for MiniLED backlighting, the proposed method is general and can be extended to any VHG system with non-ideal sources, including augmented reality waveguides, holographic data storage, and spectral filters. Future research will focus on designing microstructures specifically tailored to the angular light distribution modulated by the VHG, aiming to achieve perfect light uniformity at near-zero OD.

## Figures and Tables

**Table 1:** Parameters for transmission and reflection VHGs.

Grating Type	Grating Period	Recording Wavelength	Grating Thickness	Bragg Angle	Refractive Index Modulation
Transmission VHG	0.247 μm	532 nm	15 μm	45°	0.018
Reflection VHG	0.247 μm	532 nm	15 μm	45°	0.030

**Table 2:** Simulation angles and peak irradiance for each group.

Group	VHG	Peak Irradiance (W/cm^2^)
1	\	5.01
2	(0°, 45°)	4.96
3	(−5.98°, −51.0°)	4.46

**Table 3:** Simulation angles and peak irradiance for the dual-layer VHG group.

Group	VHG1	VHG2	Peak Irradiance (W/cm^2^)
4	(0°, 45°)	(0°, −45°)	4.96
5	(−5.98°, −51.0°)	(5.98°, 51.0°)	4.14

## Data Availability

The data that support the findings of this study are available from the corresponding authors upon reasonable request.
